# An Efficient Boron Source Activation Strategy for the Low-Temperature Synthesis of Boron Nitride Nanotubes

**DOI:** 10.1007/s40820-024-01521-2

**Published:** 2024-09-27

**Authors:** Ying Wang, Kai Zhang, Liping Ding, Liyun Wu, E Songfeng, Qian He, Nanyang Wang, Hui Zuo, Zhengyang Zhou, Feng Ding, Yue Hu, Jin Zhang, Yagang Yao

**Affiliations:** 1 National Laboratory of Solid State Microstructures, College of Engineering and Applied Sciences, Jiangsu Key Laboratory of Artificial Functional Materials, and Collaborative Innovation Center of Advanced Microstructures, Nanjing University, Nanjing, 210093 People’s Republic of China; 2 School of Electronic Information and Artificial Intelligence, Shaanxi University of Science & Technology, Xian, 710000 People’s Republic of China; 3 College of Bioresources Chemical and Materials Engineering, Shaanxi University of Science & Technology, Xian, 710000 People’s Republic of China; 4 Key Laboratory of Carbon Materials of Zhejiang Province, College of Chemistry and Materials Engineering, Wenzhou University, Wenzhou, 325000 People’s Republic of China; 5 Shenzhen Institutes of Advanced Technology, Chinese Academy of Sciences, Shenzhen, 518000 People’s Republic of China; 6 College of Chemical and Molecular Engineering, Peking University, Beijing, 100871 People’s Republic of China

**Keywords:** Boron nitride nanotubes, Low–temperature, Boron activation, Density functional theory

## Abstract

Lowering the synthesis temperature of boron nitride nanotubes (BNNTs) is crucial for their development. The primary reason for adopting a high temperature is to enable the effective activation of high-melting-point solid boron. In this study, we developed a novel approach for efficiently activating boron by introducing alkali metal compounds into the conventional MgO–B system. This approach can be adopted to form various low-melting-point AM–Mg–B–O growth systems. These growth systems have improved catalytic capability and reactivity even under low-temperature conditions, facilitating the synthesis of BNNTs at temperatures as low as 850 °C. In addition, molecular dynamics simulations based on density functional theory theoretically demonstrate that the systems maintain a liquid state at low temperatures and interact with N atoms to form BN chains. These findings offer novel insights into the design of boron activation and are expected to facilitate research on the low-temperature synthesis of BNNTs.

**Supplementary Information:**

The online version contains supplementary material available at 10.1007/s40820-024-01521-2.

## Highlights

- Developed more efficient boron activation strategies, while establishing various low-melting growth systems.
- The preparation temperature of boron nitride nanotubes has been reduced to 850 °C.

**Supplementary Information:**

The online version contains supplementary material available at 10.1007/s40820-024-01521-2.

## Graphical abstract

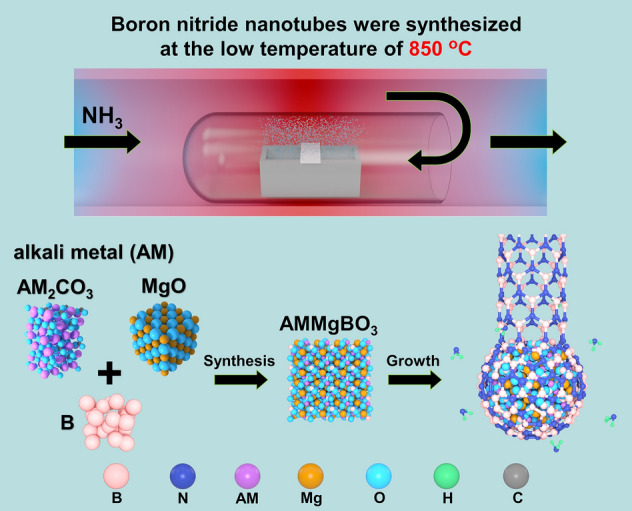

## Introduction

Boron nitride nanotubes (BNNTs) [1–3] are one-dimensional tubular nanostructures with excellent physical and chemical properties. These properties include high mechanical strength [4], low weight [5], high temperature resistance [6, 7], strong interfacial bonding [8], and electrical insulation [9]. These properties make BNNTs highly promising for various applications, including thermal interface materials, high-temperature-resistant materials, radiation shielding materials, and deep ultraviolet emitters [10–13].

Nevertheless, the high cost of BNNTs has slowed the progress of research within the aforementioned fields. Lowering the synthesis temperature helps reduce costs because high synthesis temperatures (with the current synthesis temperature being 1100–8000 °C) increase instrument complexity and energy consumption [14–31]. To enhance the economic feasibility of BNNTs, it is imperative to thoroughly research their growth mechanisms, identify the factors that necessitate a high synthesis temperature, and thereby establish ways of lowering the synthesis temperature.

The synthesis of BNNTs typically follows the vapor–liquid–solid (VLS) growth mechanism. This process involves the dissolution and precipitation of boron and nitrogen sources in a liquid catalyst. The utilization of boron sources in growth systems has been limited primarily by the high toxicity of gaseous boron sources (i.e., B_2_H_6_, B_10_H_14_, BCl_3_) and the extremely high melting point (2076 °C) of solid boron when compared with the wide use of gaseous nitrogen sources (N_2_, NH_3_). The conversion of solid B into a highly activated state demands much energy, which is a major contributing factor to the high growth temperature. Our primary task, therefore, is to explore a method of activating boron that is not only low energy but also efficient to reduce the growth temperature.

Over the years, methods of activating boron have been primarily categorized into physical and chemical techniques. Physical techniques include laser ablation [14, 15], thermal plasma [16, 17], and arc discharge [18, 19] methods. These methods use the high temperatures generated by lasers, plasma, and arc discharge to break down the boron, resulting in highly active boron atoms. These atoms then aggregate into boron droplets, which are used in BNNT synthesis. Despite their ability to produce high-quality BNNTs, physical methods have drawbacks, including the requirement for complex and expensive equipment and extremely high reaction temperatures (3500–8000 °C) (Table S1). An alternative approach that combines physical and chemical techniques for boron activation is the adoption of the ball milling and annealing method [20–22]. Mechanical ball milling reduces the size of boron such that the boron reacts with NH_3_ to form activated B–N nanoparticles. These nanoparticles subsequently crystallize into nanotubular structures during annealing. Although this method lowers the required activation temperature to 1100–1300 °C, it has limitations, including a relatively low efficiency in activating boron and a susceptibility to the deactivation of active boron (Table S1).

In comparison, boron oxide chemical vapor deposition (BOCVD) [23–31] is a promising method for boron activation in the field of chemical technology. This method involves the reaction of metal oxides (MeO_x_) with boron at high temperatures, resulting in the formation of B_x_O_y_ and metal vapor. Once the vapor pressure in the system reaches saturation, the B_x_O_y_ and metal vapor interact and condense into highly activated Me–B–O liquid particles [32–35]. These liquid particles undergo supersaturation and precipitation of B–N chains in the presence of NH_3_, resulting in the formation of BNNTs. The advantages of this method are its simple equipment, the controllability of the reaction, the high activation efficiency of the boron, and the high quality of the prepared BNNTs (Table S1).

However, the current growth temperature range (1100–1500 °C) of BOCVD is high due to the activity of the Me–B–O growth system formed. An analysis of phase diagrams reveals that compounds in the Me–B–O system containing highly catalytic metals (Me = Mg, Fe, Al, Ca) typically have high melting points, resulting in low catalytic growth activity of the system at lower temperatures (Fig. S1). The above analysis indicates the necessity of a more thorough investigation of the conventional MgO–B growth system (where a schematic diagram of the experiment is shown in Fig. S2) [36]. MgO has exceptional activation effects on B, effectively constructing the Mg–B–O growth system (where detailed experimental explanations are presented in Fig. S3). The high-melting-point compound Mg_2_B_2_O_5_ (1307 °C) [37] generated in this system has a strong catalytic capability for BNNT growth at high temperatures. However, this compound deviates from the VLS growth mechanism in that it does not liquefy at lower temperatures, which hinders BNNT growth. Therefore, it is crucial to lower the melting point of the Mg–B–O system with high catalytic activity.

As is well known, alkali metals (AMs) such as Li, Na, K, Rb, and Cs have extremely high reactivity, which is highly beneficial for activating B. Furthermore, compounds formed in the AM–B–O system typically have lower melting points, which can keep the growth system in a liquid state at reduced temperatures (Fig. S4). Therefore, in this work, we designed a method of incorporating AM compounds into the conventional MgO–B growth system. The designed method was used to establish a variety of low-melting-point AM–Mg–B–O systems, enabling efficient BNNT synthesis at a mild temperature of 950 °C and even facilitating BNNT synthesis at temperatures as low as 850 °C. Incidentally, the lowest synthesis temperature in this study is already comparable to the synthesis temperature of non-growth methods (e.g., template methods) [38, 39]. In addition, molecular dynamics simulations based on density functional theory theoretically demonstrated that the systems maintain a liquid state at low temperatures and interact with N atoms to form BN chains. This paper thus provides an innovative method for designing lower-melting-point and high-activity systems in low-temperature environments, with noteworthy implications for future research on low-temperature BNNT synthesis. As an example application, an BNNT/epoxy resin (EP) composite film, comprising 5 wt% BNNTs, exhibits remarkable thermal dissipation capabilities.

## Experimental Section

### Materials

The horizontal resistance heating furnace (GSL–1500X) was provided by Hefei Cogent Materials Technology Co., Ltd. Argon and ammonia gases were supplied by Nanjing Tezhong Gas Factory Co., Ltd. Boron powder (B), magnesium oxide (MgO), and potassium carbonate (K_2_CO_3_), lithium carbonate (Li_2_CO_3_), sodium carbonate (Na_2_CO_3_), rubidium carbonate (Rb_2_CO_3_), cesium carbonate (Cs_2_CO_3_), isopropanol (C_3_H_8_O) and ethyl acetate (C_4_H_8_O_2_) were purchased from Shanghai Aladdin Biochemical Technology Co., Ltd. Epoxy resin (EP) was supplied by Shanghai Yuanye Biotechnology Co., Ltd.

### Preparation of BNNTs

BNNTs were synthesized in a horizontal resistance heating furnace consisting of an alumina tube with a length of 60 cm and a diameter of 5 cm. The SiO_2_/Si substrates and 200 mg of MgO, K_2_CO_3_, and B precursors (molar ratio 1:1:4) were placed on the top and inside of the BN boat, respectively. The boat was positioned near the closed–end of a 20 cm long, 3 cm diameter alumina tube. This small tube was placed inside a horizontal resistance heating furnace, and the closed–end was kept in the center of the heating zone (as shown in Fig. S2). The furnace was continuously supplied with 50 standard cubic centimeters per minute (sccm) Ar and heated to the growth temperature at a rate of 10 °C min^−1^. Subsequently, the Ar was replaced with 50 sccm NH_3_ and maintained for 2 h to enable the growth of BNNTs. The resulting sample was then cooled to room temperature under Ar protection.

### Preparation of BNNT/EP Composite Films

A homogeneous solution was formed by mixing 10 mL of isopropanol, 10 mL of ethyl acetate, and 2 g of EP at 60 °C and a stirring rate of 500 r min^−1^. Then, 0.1 g of BNNTs was added to the EP solution and kept under the same stirring and temperature conditions until the solution became a viscous and homogeneous mixture. A curing agent, diethylenetriamine, was then added to the mixture in a quantity of 200 μL. After stirring for 2 min, the mixture was poured into a Teflon Petri dish and cured at 60 °C for 2 h to produce the composite film.

## Results and Discussion

### Growth Results of BNNTs in the K-Mg-B-O System

**Fig. 1:**
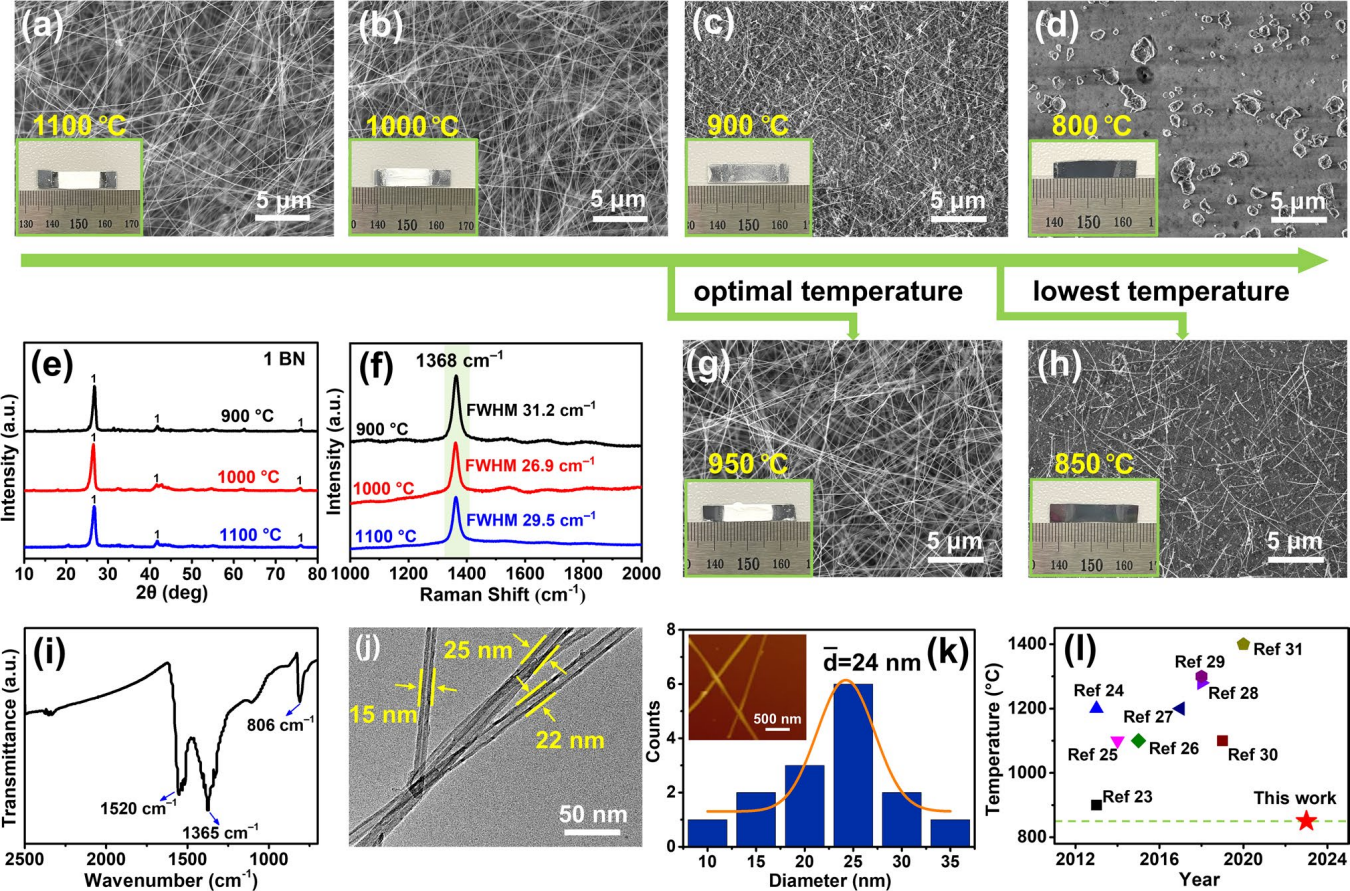
Fundamental characterization of BNNTs. SEM images of products grown on SiO_2_/Si substrates at **a** 1100 °C, **b** 1000 °C, **c** 900 °C, **d** 800 °C, **g** 950 °C, and **h** 850 °C. Inset: photograph of products formed on SiO_2_/Si substrates. **e** XRD patterns and **f** Raman spectra of products formed at 900, 1000, and 1100 °C. **i** Fourier transform infrared spectrum of products formed at 950 °C. **j** Low-magnification TEM images of the BNNTs. **k** Diameter distribution of the BNNTs (inset: typical AFM image of BNNTs dispersed on the SiO_2_/Si substrates). **l** Comparison of growth temperatures between previous reports and this work

We initially present the growth outcomes of incorporating K, an AM, into MgO–B. Optical photographs and scanning electron microscopy (SEM) images of the growth results for MgO, K_2_CO_3_, and B with a molar ratio of 1:1:4 reveal the deposition of white products, including slender BNNTs, on SiO_2_/Si substrates at growth temperatures of 1000 and 1100 °C (Fig. 1a, b). At a growth temperature of 900 °C, a minimal quantity of product was acquired on the SiO_2_/Si substrate (Fig. 1c). Nonetheless, BNNT synthesis was unsuccessful at 800 °C (Fig. 1d). X-ray diffraction (XRD) and Raman spectra analyses were conducted for the products on the SiO_2_/Si substrates obtained at growth temperatures of 900, 1000, and 1100 °C. The peaks observed in the XRD patterns correlate with distinctive signals of h-BN (JCPDS No. 73–2095), signifying that the as-grown samples have h-BN structures (Fig. 1e). The Raman spectra have a pronounced absorption band at 1368 cm^–1^, corresponding to the E_2g_ in-plane vibration mode of h-BN (Fig. 1f). The XRD patterns suggest that the products within the BN boat encompass BN (JCPDS No. 73–2095), MgO (JCPDS No. 87–0652), and a minor quantity of B_13_C_2_ (JCPDS No. 71–0108) (resulting from the reaction between CO_2_ from K_2_CO_3_ decomposition and B) (Fig. S5). This indicates that the system’s precursors undergo efficient transformation into BN even at low temperatures.

Furthermore, a more thorough temperature investigation revealed a temperature of 950 °C to be suitable for efficient growth. White material (BNNTs) was observed on the precursor material and along the inner walls of the BN boat, as illustrated in Fig. S6a. SEM images unveiled that these white products comprised an extensive region of densely packed filamentous BNNTs, having an estimated length of several micrometers to tens of micrometers (Figs. S6b and 1g). In addition, apart from XRD and Raman spectra affirming the h-BN structure of the samples cultivated at 950 °C (Fig. S6c, d), further characterization was undertaken via Fourier transform infrared spectroscopy and transmission electron microscopy (TEM) for a more comprehensive analysis. In Fig. 1i, there are three absorption bands at approximately 1520, 1365, and 806 cm^–1^. The absorption peak at 806 cm^–1^ corresponds to a B–N–B bending vibration parallel to the c-axis whereas the absorption peak at 1365 cm^–1^ corresponds to a B–N stretching vibration perpendicular to the c-axis. Both of these peaks are characteristic of BN. The absorption peak at 1520 cm^–1^ is associated with the vibration of the BN skeleton along the tangential direction of the nanotubes, which is a distinct characteristic of BNNTs. The TEM images reveal that the samples have elongated and straight hollow tubular structures, with diameters ranging from 15 to 25 nm (Fig. 1j). The interplanar spacing of the wall, which is characteristic of a *d*_(002)_ spacing of h-BN, is approximately 0.34 nm (Fig. S6e). The BNNTs were then dispersed onto SiO_2_/Si substrates to obtain diameter statistics. SEM images (Fig. S6f) and atomic force microscopy (AFM) images (Fig. 1k** inset**) depict the relatively isolated and well-dispersed nature of the BNNTs on a SiO_2_/Si substrate. The primary diameter distribution of the BNNTs, spanning from 10 to 35 nm and averaging 24 nm, is illustrated in Fig. 1k. All of the above results substantiate the effective synthesis of high-quality BNNTs at a modest temperature of 950 °C. Finally, and most surprisingly, the system accomplished BNNT synthesis at a minimum temperature of 850 °C (Fig. 1h). A comparison of the lowest growth temperature (850 °C) in this study with previously reported temperatures (Fig. 1l) reveals the achievement of this study (refer to Table S2 for detailed information).

### Analysis of the Reasons for Low-Temperature Synthesis in the K-Mg-B-O System

**Fig. 2:**
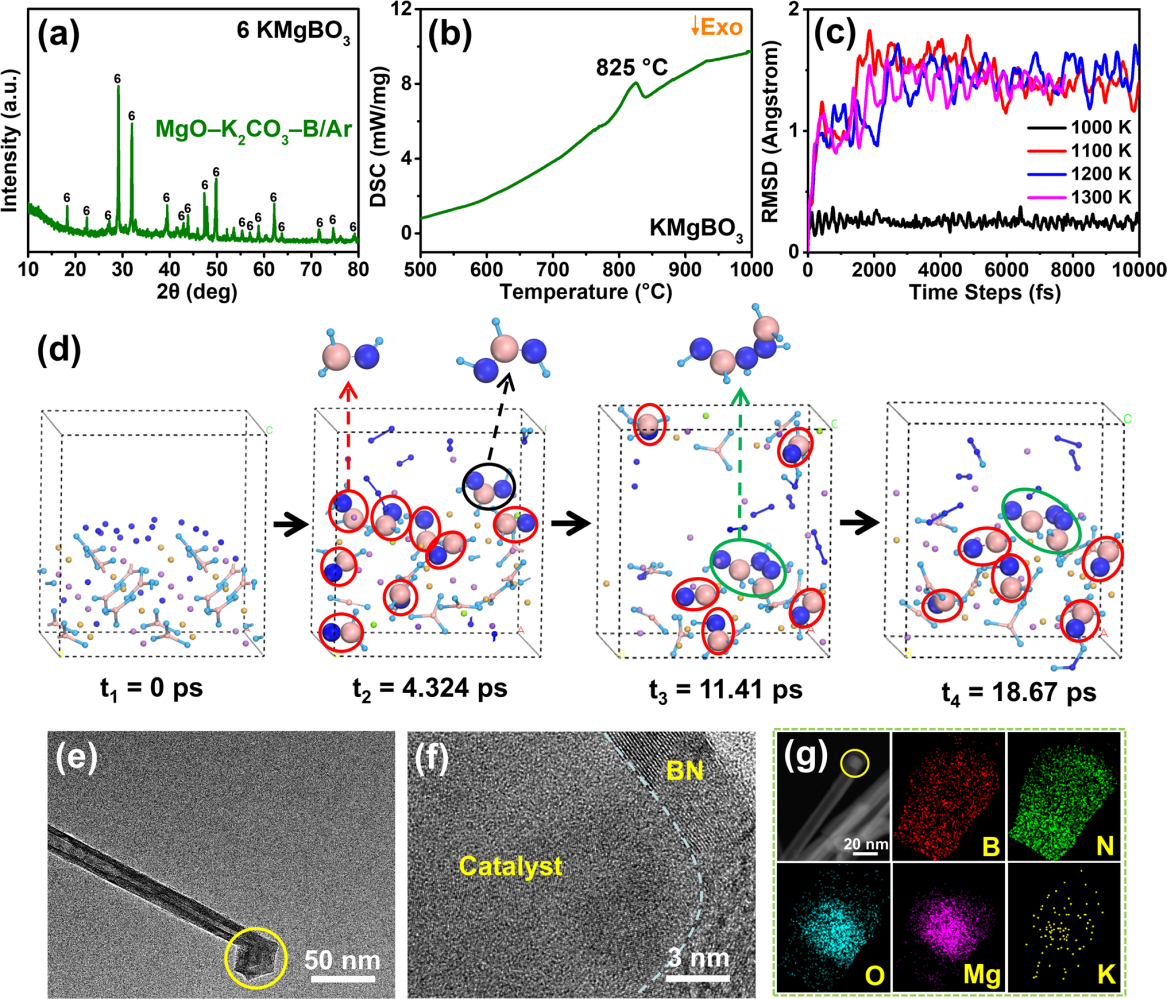
Experimental and theoretical analysis of the growth mechanism. **a** XRD patterns of products formed from MgO–K_2_CO_3_–B under Ar at 850 °C. **b** DSC curves of KMgBO_3_. **c** RMSD of the atoms of KMgBO_3_ in MD simulations at different temperatures. **d** Formation of h-BN on the surface of KMgBO_3_ observed in the MD simulation at 1100 K. Yellow: Mg, Purple: K, Light blue: O, Pink: B, Dark blue: N. **e** Low- and **f** high-magnification and **g** energy-dispersive X-ray spectroscopy mapping of the tip of the as-grown BNNTs

Subsequently, the growth mechanism of the system was investigated. The initial stage comprised annealing MgO, K_2_CO_3_, and B under Ar at 850 °C to investigate the authentic active constituents of the precursors during the growth process. The XRD pattern indicates that the product that formed after annealing the precursors was KMgBO_3_ (ICSD No. 174336) (Fig. S10a) (Fig. 2a) [40, 41]. The melting point of this compound was then investigated adopting differential scanning calorimetry (DSC). Figure 2b shows a clear endothermic peak at 825 °C, corresponding to the melting point of KMgBO_3_. We consider that this compound has a reduced melting point, rendering it a pivotal determinant for BNNT growth at reduced temperatures. For theoretical support, density-functional-theory-based molecular dynamics (DFT-MD) simulations were conducted to study the melting of KMgBO_3_ and the mechanism of BNNT growth from liquid KMgBO_3_ via a VLS mechanism at low temperature. The root mean square deviation (RMSD) can be used as an indicator of the dissolution of alloys [42–44] and clusters [45, 46]. We first ran DFT-MD simulations to test the states of a KMgBO_3_ slab at different temperatures and calculated the RMSD (Fig. 2c). Snapshots of MD simulations taken at 10 ps (Fig. S7) clearly show that the KMgBO_3_ slab melted at ~ 1100 K, which is in good agreement with the DSC data and the lowest temperature for BNNT growth, namely ~ 850 °C. To further simulate the growth of BNNT catalyzed by KMgBO_3_, MD simulations were performed at 1100 K for a total duration of 20 ps, with a time step of 1 fs. By adding N atoms to the surface of liquid KMgBO_3_, we found that N atoms were intercalated into the B–O bonds of BO_3_^3–^. Snapshots of the MD trajectory are presented in Fig. 2d and Movie S1. The intercalation of N atoms led to the formation of N–B pairs (red circles in Fig. 2d), N–B–N trimers (black circles), and N–B–N–N–B (green circles) short chains at 1100 K, suggesting that liquified KMgBO_3_ promoted the formation of h-BN on its surface. Remarkably, these B–N chains remained intact throughout the dynamics simulation (Movie S1), indicating that the B–N bonds were more favorable than the B–O bonds. Although we could not simulate the formation of a BNNT due to the huge computational costs of the DFT-MD simulations, these results clearly show that the nucleation of BN chains on the KMgBO_3_ surface is preferred and that the speculated VLS mechanism [47] of BNNT growth is thus reasonable. This finding has been confirmed by other studies [48–50]. To acquire conclusive proof of the catalytic role of KMgBO_3_ in BNNT growth, TEM characterization was performed for the BNNT tips. Figure 2e, f clearly shows that the catalytic particle resides at the tip’s central point, encircled by layers of BN. This indicates that the BNNT grows from the catalyst particles, which is consistent with the VLS mechanism. Energy-dispersive X-ray spectroscopy (EDX) was adopted to investigate the BNNT tips. Elemental maps disclose the uniform distribution of B and N across the tip, with O and Mg being concentrated at the catalyst (Fig. 2g). In addition, the presence of K elements at the tip confirms that K has dissolved into the MgO–B system.

### Growth Results of the K-B-O System and the AM-B-Mg-O System

To investigate whether the K–B–O system catalyzes the formation of nanotubes, we studied the activation effect of K_2_CO_3_ on B at different temperatures and the growth results of K_2_CO_3_ and B (with a molar ratio of 1:2 for K_2_CO_3_ and B). The results reveal that a considerable quantity of white product formed on the SiO_2_/Si substrate at growth temperatures of 900, 1000, and 1100 °C. SEM images show that the products obtained at all three temperatures had a flake-like structure inherent to BN (Fig. S8a–c). Furthermore, the XRD pattern of the products within the BN boat demonstrated a precursor reaction with NH_3_, yielding h-BN and a minor portion of B_13_C_2_ (JCPDS No. 71–0108) (Fig. S8d). Subsequently, K_2_CO_3_ and B were subjected to annealing at 950 °C to investigate their growth mechanism. The XRD pattern in Fig. S8e indicates the precursor’s conversion into K_2_B_4_O_7_ (JCPDS No. 70–1494), having a melting point of 780 °C [51]. We propose that in this growth process, the elevated oxygen transfer propensity of K_2_O (as per the Ellingham diagram) [52] enhances B activation, resulting in the creation of low-melting-point K_2_B_4_O_7_. However, the pronounced reactivity of K renders it incapable of stable existence within the system, precluding its role in nucleation catalysis. This leads to a direct reaction between B_x_O_y_ within K_2_B_4_O_7_ and NH_3_, yielding BN nanosheets [35].

**Fig. 3:**
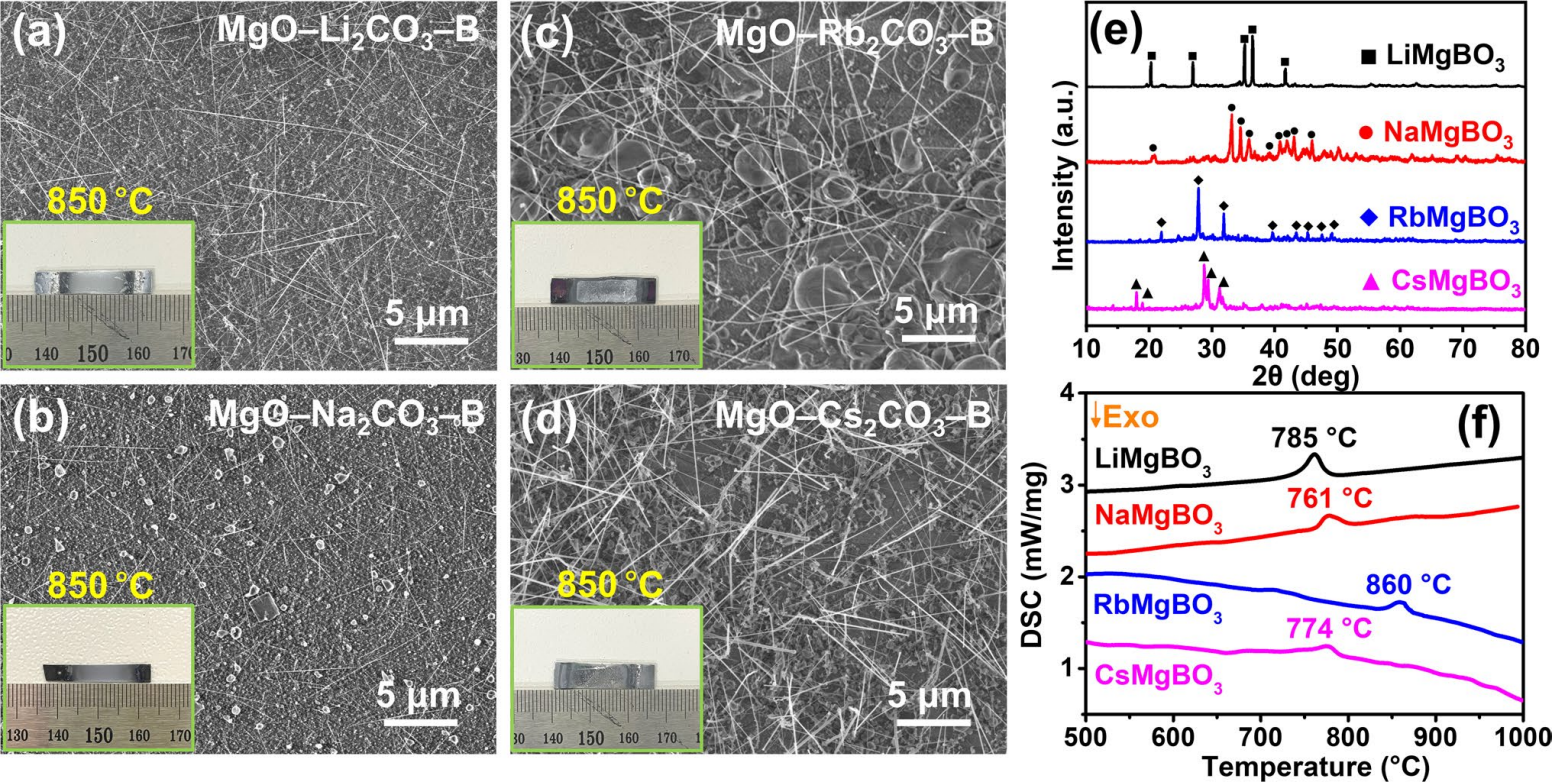
Various AMs used to grow BNNTs. SEM images of BNNTs grown on SiO_2_/Si substrates from **a** MgO–Li_2_CO_3_–B, **b** MgO–Na_2_CO_3_–B, **c** MgO–Rb_2_CO_3_–B, and **d** MgO–Cs_2_CO_3_–B at 850 °C. **e** XRD patterns of products formed from MgO–Li_2_CO_3_–B, MgO–Na_2_CO_3_–B, MgO–Rb_2_CO_3_–B, and MgO–Cs_2_CO_3_–B under Ar at 850 °C. **f** DSC curves of LiMgBO_3_, NaMgBO_3_, RbMgBO_3_, and CsMgBO_3_

To investigate the effects of other AMs on the system, experiments were conducted by adding Li_2_CO_3_, Na_2_CO_3_, Rb_2_CO_3_, and Cs_2_CO_3_ separately to the MgO–B system (MgO:AM_2_CO_3_:B with a molar ratio of 1:1:4). SEM images show that all systems with Li, Na, Rb, and Cs grew extensive BNNTs at 950 °C (Fig. S9), and all four systems grew BNNT at a lower temperature of 850 °C (Fig. 3a–d). Subsequently, we annealed the four precursors in an Ar environment. XRD patterns revealed that, following annealing at 850 °C, the four precursors produced LiMgBO_3_ (JCPDS No. 79–1996) (Fig. S10b) [53], NaMgBO_3_ (ICSD No. 249567) (Fig. S10c) [54], RbMgBO_3_ (Fig. S10d) [55], and CsMgBO_3_ (Fig. S10e) [55] (Fig. 3e). We conducted DSC tests on these four products. Figure 3f shows that the melting points of LiMgBO_3_, NaMgBO_3_, RbMgBO_3_, and CsMgBO_3_ are 785, 761, 860, and 774 °C, respectively. These results clearly indicate that AM readily dissolves into MgO–B, creating low-melting-point and highly catalytic AM–Mg–B–O systems and thus facilitating the low-temperature synthesis of BNNTs.

### Exploration of BNNT Properties and Applications

**Fig. 4:**
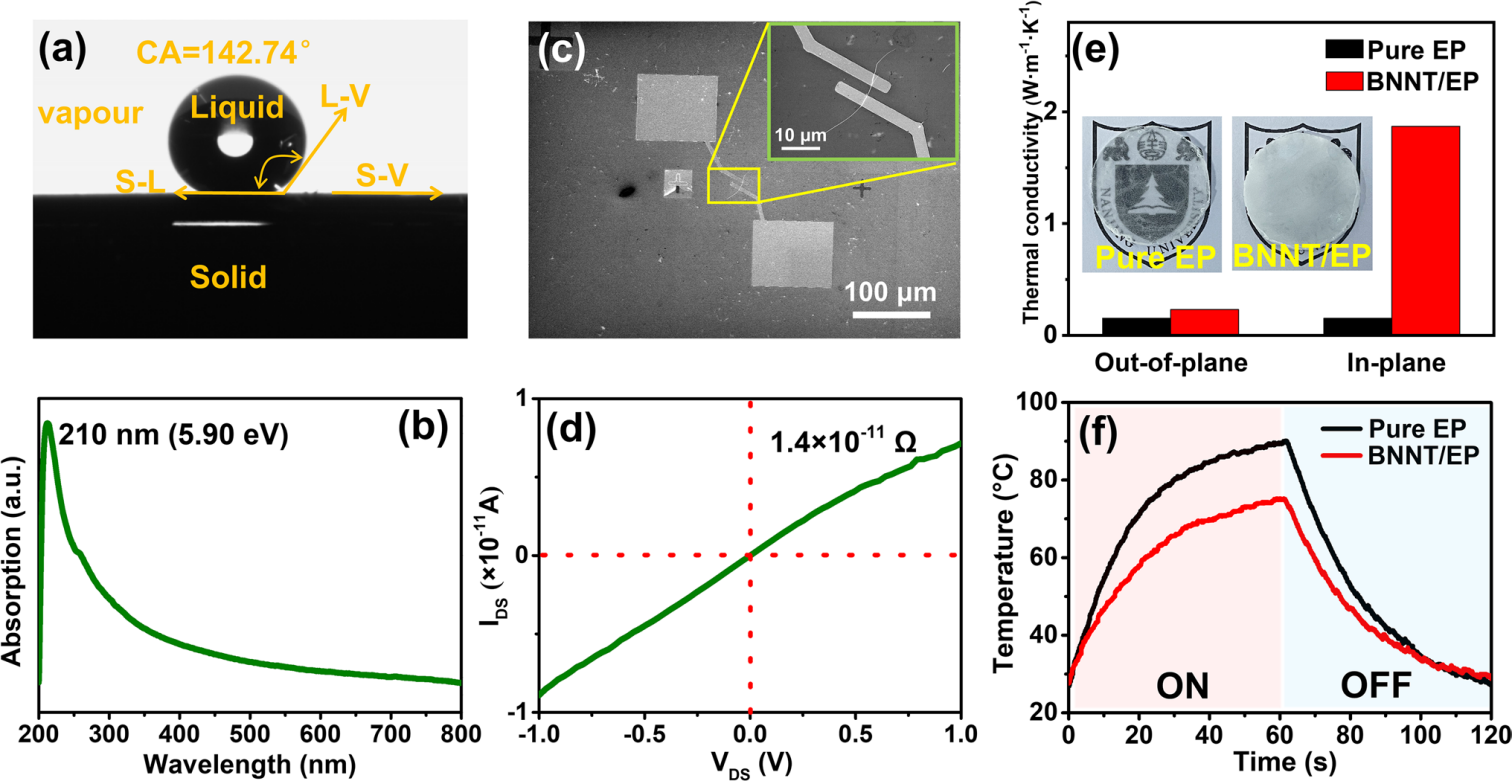
Properties and Applications of BNNTs. **a** Photographs of a CA measurement and **b** ultraviolet–visible absorption spectrum of as-grown BNNT-coated SiO_2_/Si. **c** SEM image and **d** typical I_DS_–V_DS_ curve of a single BNNT-based device. **e** In-plane and out-of-plane thermal conductivity. Inset: photograph of pure EP film and BNNT/EP composite film. **f** Surface temperature evolution over time of the pure EP film and BNNT/EP composite film

We next explored the characteristics of the synthesized BNNTs. Thermogravimetric analysis indicates that carbon nanotubes (CNTs, Shanghai Aladdin Bio-Chem Technology Co., Ltd.) underwent oxidation at 500 °C, whereas the weight of BNNTs varied minimally within the temperature range of 25 to 1000 °C, signifying thermal stability superior to that of CNTs (Fig. S11a). We adopted the drop-casting method to measure the water contact angle (CA) of the BNNTs grown on the SiO_2_/Si substrate. As is well-known, the CA is defined as the angle between the tangent to the gas–liquid interface at the triple point of gas, liquid, and solid phases and the solid–liquid boundary line on the liquid side. The size of the CA represents the degree of wettability. When the CA is 0°, it indicates complete wetting. When the CA is less than 90°, it indicates partial wetting or wetting. When the CA is greater than 90°, it indicates non-wetting. After a water droplet made contact with the SiO_2_/Si substrate, CA measurements were collected every 1 s for 10 s. The average CA was calculated as approximately 142.74°, indicating the stable hydrophobic nature of the BNNTs (Fig. 4a). Moreover, the BNNTs had consistent hydrophobic characteristics across a broad pH range spanning from 2 to 14 (Fig. S11b). These findings imply that the non-wettability of BNNTs remains impervious to potent acidic and alkaline conditions, making BNNTs highly promising for water-resistant coatings. An ultraviolet test was conducted on a suspension of BNNTs in ethanol. Figure 4b shows an absorption peak at approximately 5.9 eV (~ 210 nm), which is associated with the optical band gap of BNNTs and implies potential utility in ultraviolet-range photovoltaic devices. To examine the electrical traits of the samples, a single-BNNT-based device, featuring Cr (10 nm)/Au (80 nm) electrodes, was fabricated adopting electron beam lithography (Fig. 4c). A typical drain–source current versus drain–source voltage (*I*_DS_–*V*_DS_) curve of a single BNNT showed a current of 7 × 10^–12^ A and a resistance of 1.4 × 10^11^ Ω at *V*_DS_ = 1 V, demonstrating the BNNT’s good insulating property at room temperature due to the wide energy band gap of the nanomaterials (Fig. 4d).

The unique combination of high thermal conductivity and aspect ratio makes the BNNT an excellent filler for thermally conductive composites. A colorless and transparent pure EP film and white BNNT/EP composite film containing 5 wt% BNNT were separately fabricated using a solution method (Fig. 4e** inset**). SEM was conducted to characterize the fracture surface morphology of the composite materials. Figure S12 shows the uniform dispersion of interconnecting BNNTs throughout the composite. The XRD pattern of the pure EP film had wide diffraction peak dispersion between 15 and 23° [56], whereas the BNNT/EP composite film had a sharp peak at 26.8° corresponding to the (002) plane of h-BN (JCPDS No. 73–2095) in addition to the characteristic peaks of pure EP film (Fig. S13). The thermal conductivity of the sample was determined using the laser flash method. Figure 4e shows that the thermal conductivity of the 5 wt% BNNT/EP composite film increased to nearly 1.87 W m^−1^ K^−1^ (in plane) and 0.231 W m^−1^ K^−1^ (out of plane), approximately 12.2 times and 1.5 times higher than the values for the pure EP film, respectively. The BNNT/EP composite film was used as a thermal interface material for light-emitting diode (LED) chip heat dissipation, and the surface temperature changes of the LED chip were directly observed using an infrared thermal imager. Figures 4f** and **S14, respectively, show the variation of the core temperature of the LED chip with the running time and an infrared thermal image. During the 60 s that the LED chip was turned on, the center temperature of the BNNT/EP composite film was consistently 10–15 °C lower than that of the pure EP film. After turning off the LED chip, the core temperature of the BNNT/EP composite film dropped to 47.7 °C within 20 s, demonstrating the excellent heat dissipation ability of the BNNT/EP composite film. These findings demonstrate the application potential of BNNTs in thermal management.

## Conclusions

In summary, we developed an effective strategy for activating boron by introducing AM compounds into the traditional MgO–B system. We thus formed several innovative AM–Mg–B–O systems with low melting points and strong catalytic capabilities. All of the AM systems synthesized BNNTs at a low temperature of 850 °C. MD simulations indicated that the representative compound KMgBO_3_ can liquefy at ~ 1100 K and react with N to produce BN chains. This outcome theoretically demonstrates the practicality of the systems for the synthesis of BNNTs at low temperatures. We believe that this work has implications for future research on the low-temperature synthesis of BNNTs. In addition, we prepared BNNT/EP composite films having a thermal conductivity 12.2 times that of pure EP films and excellent heat dissipation performance.

## Supplementary Information

Below is the link to the electronic supplementary material.

Supplementary file1 (AVI 33307 kb)

Supplementary file2 (DOCX 25530 kb)
