# Supplementary material for: An Efficient Boron Source Activation Strategy for the Low-Temperature Synthesis of Boron Nitride Nanotubes
Source: Nanomicro Lett. 2024 Sep 27;17:25. doi: 10.1007/s40820-024-01521-2 (PMC11436672; doi:10.1007/s40820-024-01521-2)
Supplement: Supplementary file 2 — Supplementary file2 (DOCX 25530 kb) [file 40820_2024_1521_MOESM2_ESM.docx]

Supporting Information for

**An Efficient Boron Source Activation Strategy for the**

**Low-Temperature Synthesis of Boron Nitride Nanotubes**

Ying Wang^1,#^, Kai Zhang^1,#^, Liping Ding^2,#^, Liyun Wu^1^, Songfeng E^3^, Qian He^1^, Nanyang Wang^1^, Hui Zuo^4^, Zhengyang Zhou^1^, Feng Ding^5^, Yue Hu^4,^ *, Jin Zhang^6,^ *, Yagang Yao^1,^ *

^1^ National Laboratory of Solid State Microstructures, College of Engineering and Applied Sciences, Jiangsu Key Laboratory of Artificial Functional Materials, and Collaborative Innovation Center of Advanced Microstructures, Nanjing University, Nanjing 210093, P. R. China

^2^ School of Electronic Information and Artificial Intelligence, Shaanxi University of Science & Technology, Xian 710000, P. R. China

^3^ College of Bioresources Chemical and Materials Engineering, Shaanxi University of Science & Technology, Xian 710000, P. R. China

^4^ Key Laboratory of Carbon Materials of Zhejiang Province, College of Chemistry and Materials Engineering, Wenzhou University, Wenzhou 325000, P. R. China

^5^ Shenzhen Institutes of Advanced Technology, Chinese Academy of Sciences, Shenzhen 518000, P. R. China

^6^ College of Chemical and Molecular Engineering, Peking University, Beijing 100871, P. R. China

^#^Ying Wang, Kai Zhang and Liping Ding contributed equally to this work.

*Corresponding authors. E-mail:, [yuehu@wzu.edu.cn](mailto:yuehu@wzu.edu.cn) (Yue Hu); [jinzhang@pku.edu.cn](mailto:jinzhang@pku.edu.cn) (Jin Zhang); [ygyao2018@nju.edu.cn](mailto:ygyao2018@nju.edu.cn) (Yagang Yao)

**S1 Experimental Section**

**S1.1 Calculation Methods**

All the density functional theory (DFT)–molecular dynamic (MD) were implemented in the Vienna Ab initio Simulation Package (VASP) [S1, S2]^.^ The projector–augmented wave (PAW) method [3–4] was used to describe the interaction of valence electrons–ion cores, and the Perdew–Burke–Ernzerhof (PBE) [3] exchange–correlation functional was adopted for the interaction between valence electrons. We used a plane wave basis set with a cutoff energy of 450 eV in the simulations. The convergence criteria for energy and force were set at 10^–5^ eV and 10^–2^ eV/Å, respectively. The MD simulations of KMgBO_3_–catalyzed the nucleation of BN nanotube were performed at a temperature of 1100 K. In addition, an NVT ensemble and a 1.0 fs time step were employed.

To illustrate the dissolvation of KMgBO_3_, we calculated the Root Mean Square Deviation (RMSD) of KMgBO_3_ systems using the formula:

$RMSD=\sqrt{\frac{\sum_{\text{i}=1}^{N_{atom}} \left(r_{i}\left(t_{1}\right)-r_{i}\left(t_{2}\right)\right)^{2}}{N_{atom}}}$ (S1)

where r_i_(t_1_) and r_i_(t_2_) are the positions of any atom i in the system at the time of t_1_ and t_2_, respectively. N_atom_ is the total number of atoms.

**S1.2 Characterization of BNNTs**

The morphology observation of BNNTs is conducted by Scanning electron microscopy (SEM, Hitachi SU8100), and Transmission electron microscopy (TEM, Tecnai F20 and Hitachi HT7820) and tapping–mode atomic force microscope (AFM, NanoScope IIIa, Veeco Co). Composition of as–obtained sample investigated by the X–ray diffractometer (Cu Kα, λ = 1.54 Å; LabX XRD–6000), Raman spectroscopy (HORIBA Scientific, 532–nm laser) and FTIR spectroscopy (Bruker Vertex70V+Hyperion 2000). Properties of BNNTs tested via Thermogravimetric analysis (TGA, PerkinElmer STA 6000), UV–visible absorption spectra (Shimadzu UV–2700), Water contact angle (CA, SDC100, Dongguan Zui Ding Precision Instruments Co., Ltd.), Keithley 4200–SCS semiconducting characterization system. Differential scanning calorimetry (DSC, Germany Netzsch STA 449 F3). The thermal diffusivity and the heat dissipation performancewas measured using the laser flash method (LFA 447, NETZSCH, Germany) and infrared thermograph (FOTRIC–345, China), respectively.

**S2 Supplementary Figures and Tables**

**Table S1** Boron source activation technology

| **Method for activating boron source** | | **Catalyst and boron source** | **References in the article** | **Growth Temperature (°C)** | **The principle of active boron production** | **Advantages and disadvantages** |
| --- | --- | --- | --- | --- | --- | --- |
| **physical technology** | laser ablation | c-BN, B | [14, 15] | 4000**–**5000 | high temperature generated by laser ablation | 1. high-quality BNNTs  2. complex and expensive equipment  3. extremely high reaction temperatures |
|  | thermal plasma | B, h-BN | [16, 17] | 8000 | high temperatures and plasma |  |
|  | arc discharge | YB_6_, B | [18, 19] | ＞3500 | high temperature generated by arc discharge |  |
| **combining physical and chemical technology** | ball milling and annealing | B | [20–22] | 1100–1300 | mechanical ball milling can reduce the size of B and enable it to react with NH_3_ to form activated B-N nano particles. These particles are then crystallized into nanotubular structures through annealing. | 1. low efficiency in activating the boron source  2. easy deactivation of active boron |
| **chemical technology** | boron oxide chemical vapor deposition (BOCVD) | 1. MeO_x_   Me= Mg, Fe, Li, Ca | [23–31] | 1100–1500 | metal oxides (MeO_x_) react with B at high temperatures to produce B_x_O_y_ and metal vapor. When the vapor pressure in the system reaches saturation, B_x_O_y_ and metal vapor interact and condense into highly activated Me-B-O liquid particles. | 1. simple equipment  2. controllable reaction  3. high activation efficiency of boron source  4. high quality of the prepared BNNTs  5. relatively high growth temperature |
| **our technology** | developedBOCVD | B,  AM_2_CO_3_, MgO | this work | expected temperature <1000 | AM can reduce the melting point of the Mg-O system. | 1. more efficient activated boron source  2. AM-Mg-B-O systems with low melting points and high catalytic activity. |


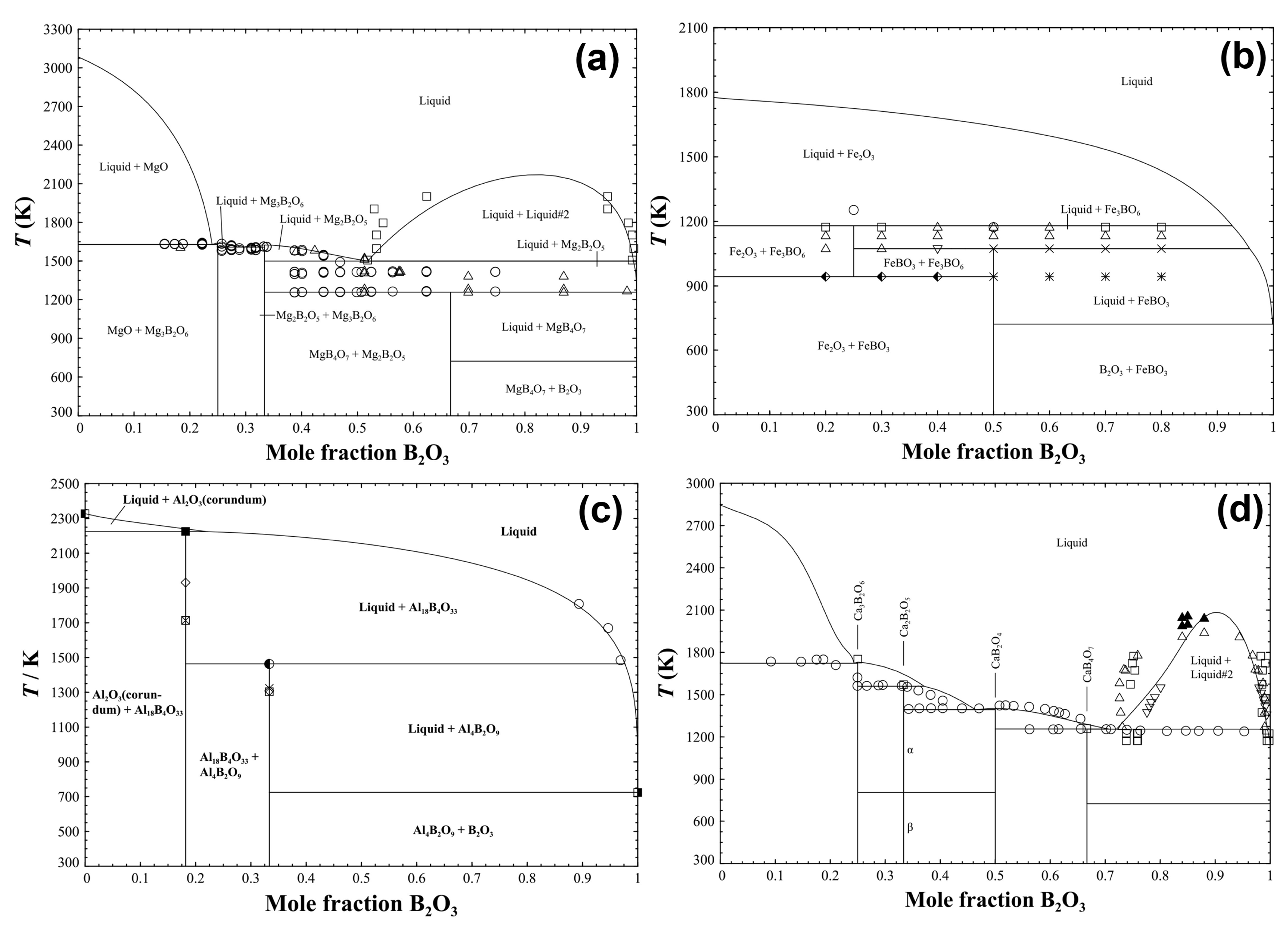


**Fig. S1** The phase diagram of B_2_O_3_ and (**a**) MgO, (**b**) Fe_2_O_3_, (**c**) Al_2_O_3_, and (**d**) CaO, respectively [S5]


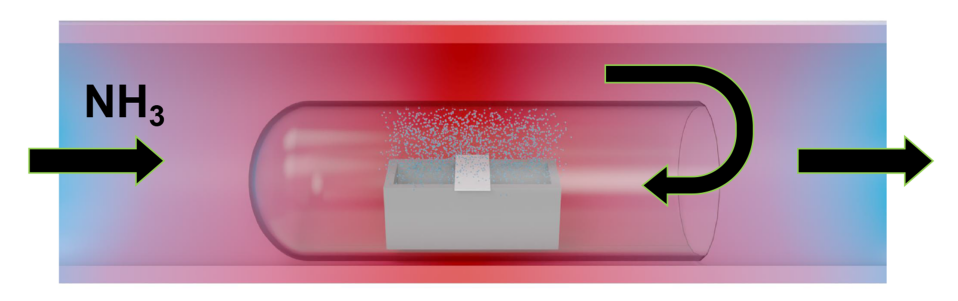


**Fig. S2** Schematic illustration of the experimental equipment
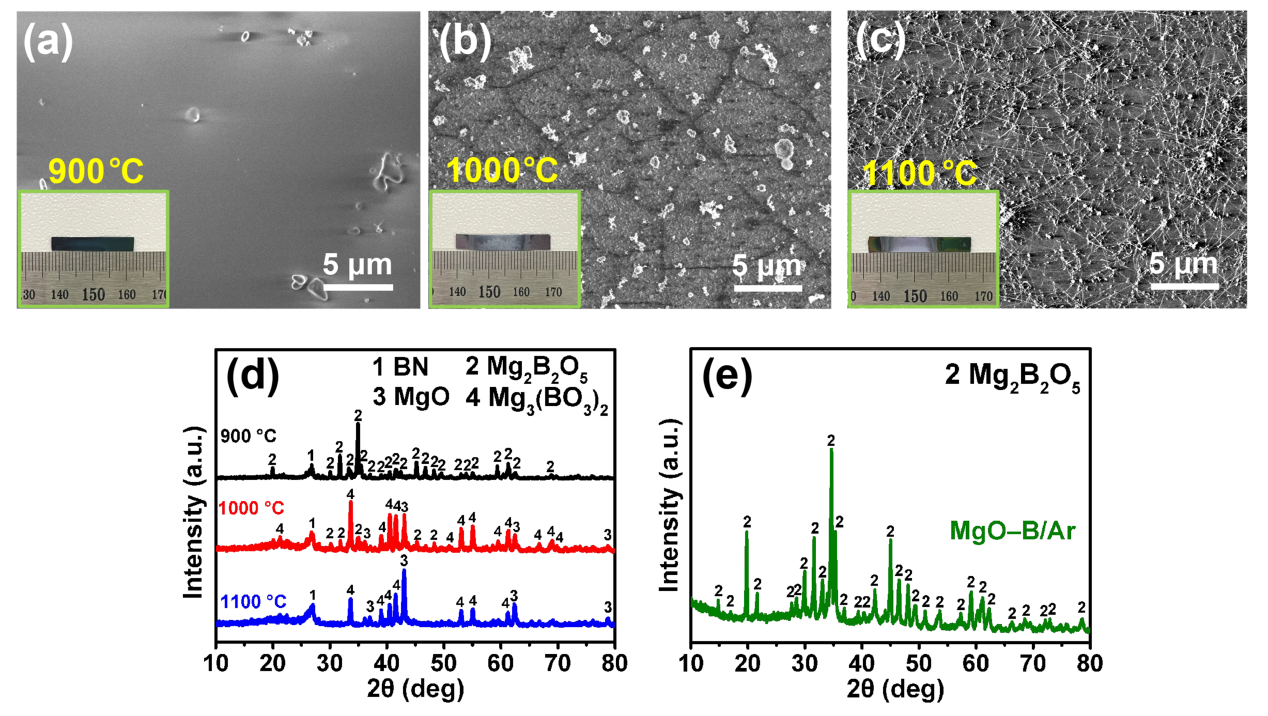


**Fig. S3** SEM images of the products grown on the SiO_2_/Si substrates from 1:2 molar ratios of MgO:B at (**a**) 900 °C, (**b**) 1000 °C and (**c**) 1100 °C. (**d**) XRD patterns of products formed in a BN boat from 1:2 molar ratios of MgO:B. (**e**) XRD patterns of products formed from 1:2 molar ratios of MgO:B under Ar at 850 °C

Data analysis: The growth experiments using MgO and B with a molar ratio of 1:2 revealed that at temperatures of 900 °C and 1000 °C, no white precipitate formed on the SiO_2_/Si substrate. SEM images also indicate the absence of BNNTs (**Fig.** **S3a and S3b**). Only at 1100 °C, a small amount of white product and a few slender BNNTs were observed on the SiO_2_/Si substrate (**Fig. S3c**). XRD pattern demonstrated that the product in the BN boat comprised of BN (JCPDS No. 73–2095), Mg_2_B_2_O_5_ (JCPDS No. 73–2232), MgO (JCPDS No. 87–0652), and Mg_3_(BO_3_)_2_ (JCPDS No. 75–1807) (**Fig. S3d**). Subsequently, the MgO and B precursors with a molar ratio of 1:2 were annealed at 850 °C under an Ar flow rate for further analysis. The XRD pattern indicated that the MgO and B precursors had transformed into high melting point Mg_2_B_2_O_5_ (1307 °C) before reacting with ammonia (**Fig. S3e**). These findings highlight the favorable activation effect of MgO on B, and the resulting Mg_2_B_2_O_5_ compound demonstrates strong catalytic ability for nanotube growth. However, this compound does not liquefy at low temperatures, making it unsuitable for the VLS growth of BNNTs at low temperatures.


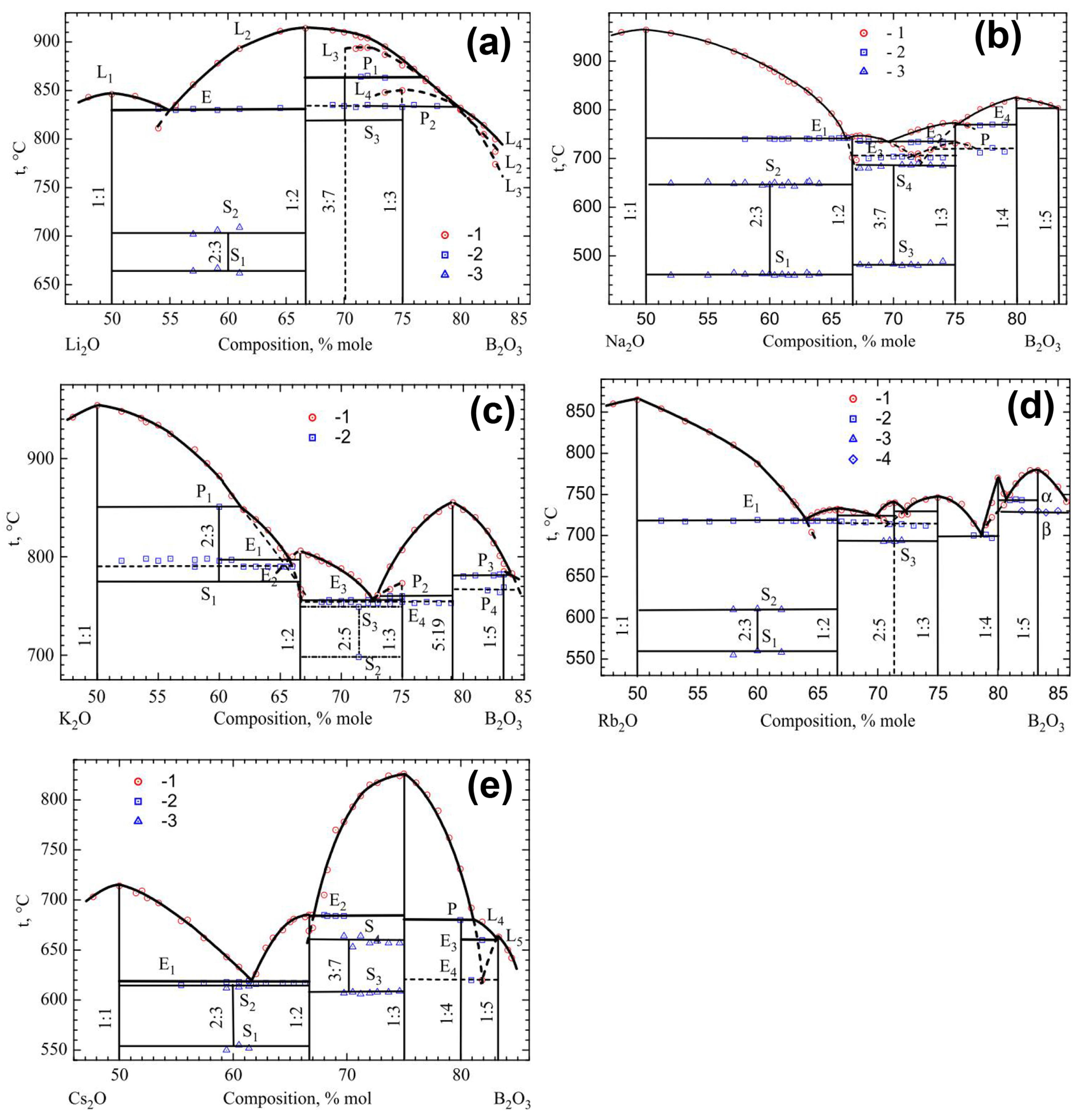


**Fig. S4** The phase diagram of B_2_O_3_ and (**a**) Li_2_O, (**b**) Na_2_O, (**c**) K_2_O, (**d**) Rb_2_O, and (**e**) Cs_2_O, respectively [S6]


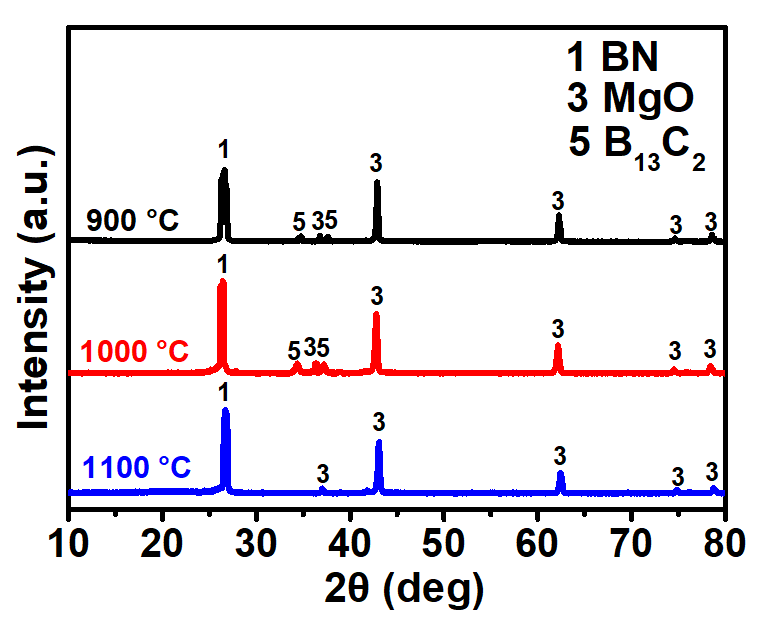


**Fig. S5** XRD patterns of products formed in a BN boat from 1:1:4 molar ratio of MgO:K_2_CO_3_:B at 900 °C, 1000 °C and 1100 °C


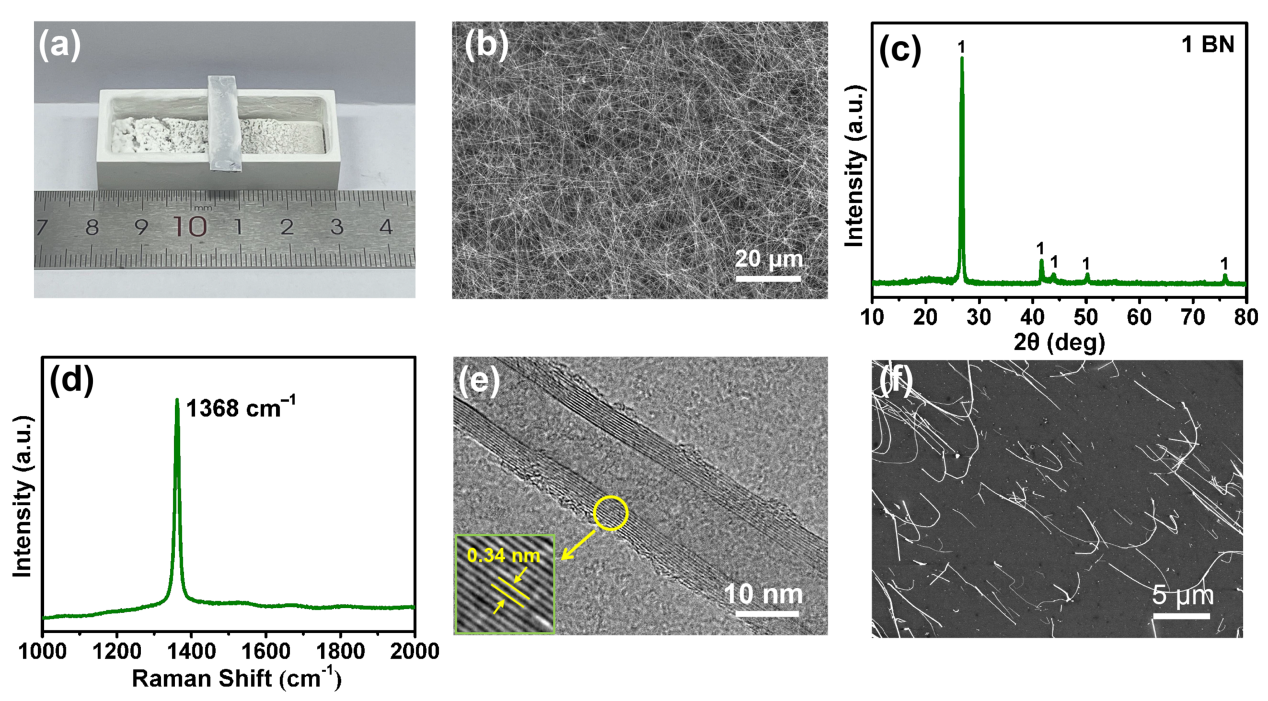


**Fig. S6** (**a**) A photograph of the precursor materials in the BN boat. (**b**) SEM images of the products grown on the SiO_2_/Si substrates from 1:1:4 molar ratios of MgO:K_2_CO_3_:B at 950 °C. (**c**) XRD pattern and (**d**) Raman spectrum of products formed in the SiO_2_/Si substrates from 1:1:4 molar ratio of MgO:K_2_CO_3_:B at 950 °C. (**e**) High–magnification TEM images of the BNNTs. (**f**) SEM image of BNNTs dispersed on the SiO_2_/Si substrates.

**Table S2** Catalytic materials and temperatures used in CVD methods

| **Precursors** | **Growth Temperature (°C)** | **Catalyst** | **Year** | **References in the article** |
| --- | --- | --- | --- | --- |
| B, Fe_2_O_3_ | 900–1400 | Fe | 2013 | [23] |
| B, Li_2_O | 1200 | Li | 2013 | [24] |
| B, V_2_O_5_, Fe_2_O_3_  B, V_2_O_5_, Ni_2_O_3_ | 1100 | V,Fe/V,Ni | 2014 | [25] |
| B, MgO, γ–Fe_2_O_3_ | 1100–1200 | Mg, Fe | 2015 | [26] |
| B/B_2_O_3_,NiY | 1200 | NiY | 2017 | [27] |
| B/(NH_4_)_10_W_12_O_41_ · xH2O | 1280 | W | 2018 | [28] |
| B_2_O_3_ and Li_3_N | 1300 | Li | 2018 | [29] |
| Mg,B_2_O_3_,H_3_BO_3_ | 1100–1400 | Mg | 2019 | [30] |
| B,MgO, MgB_2_ | 1400 | Mg | 2020 | [31] |
| B,MgO, AM_2_CO_3_ | 850–1100 | Mg, AM | 2023 | This work |


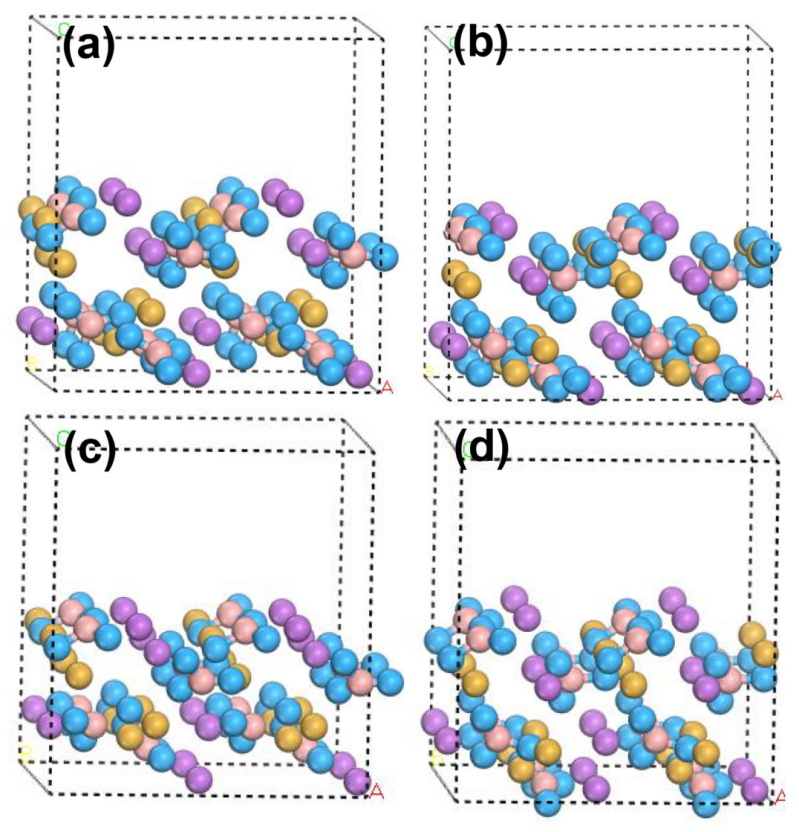


**Fig. S7** The snapshots taken at 10 ps of MD simulations at different temperatures (**a**) 1000 K, (**b**) 1100 K, (**c**) 1200 K and (**d**) 1300 K. Yellow: Mg, Purple: K, Light blue: O, Pink: B, Dark blue: N


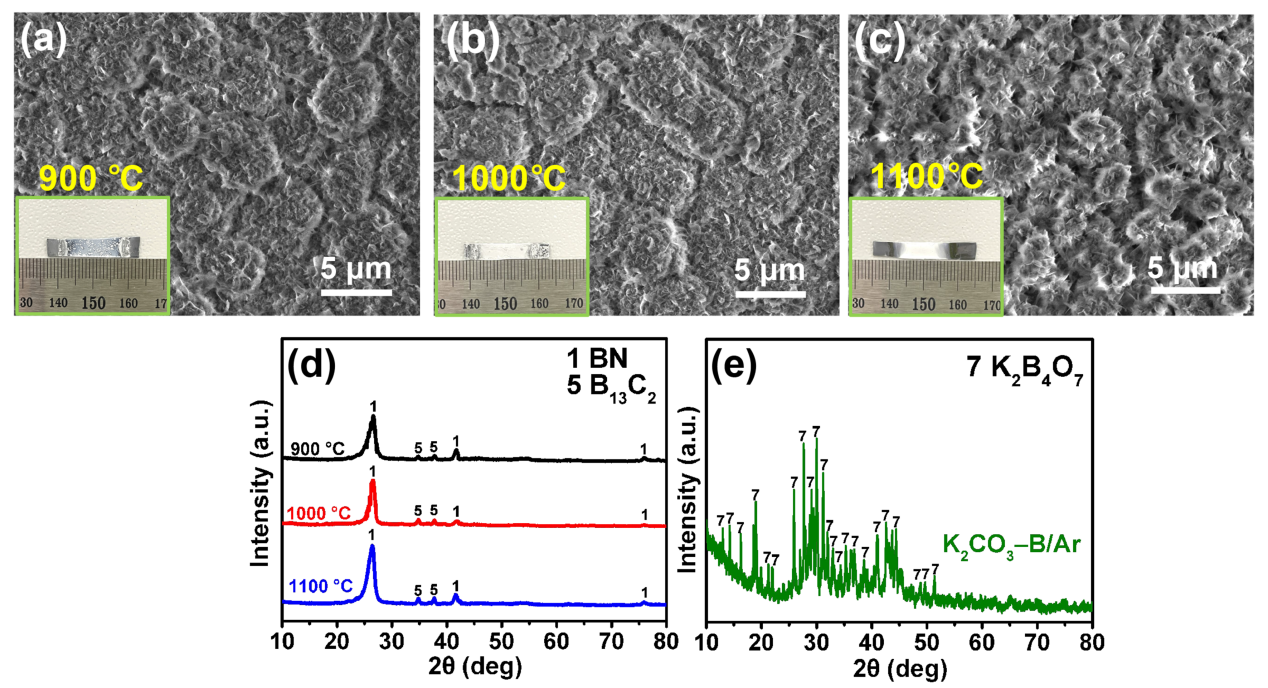


**Fig. S8** SEM images of the products grown on the SiO_2_/Si substrates from 1:2 molar ratios of K_2_CO_3_:B at (**a**) 900 °C, (**b**) 1000 °C and (**c**) 1100 °C. (**d**) XRD patterns of products formed in a BN boat from 1:2 molar ratios of K_2_CO_3_:B. (**e**) XRD patterns of products formed from 1:2 molar ratios of K_2_CO_3_:B under Ar at 850 °C


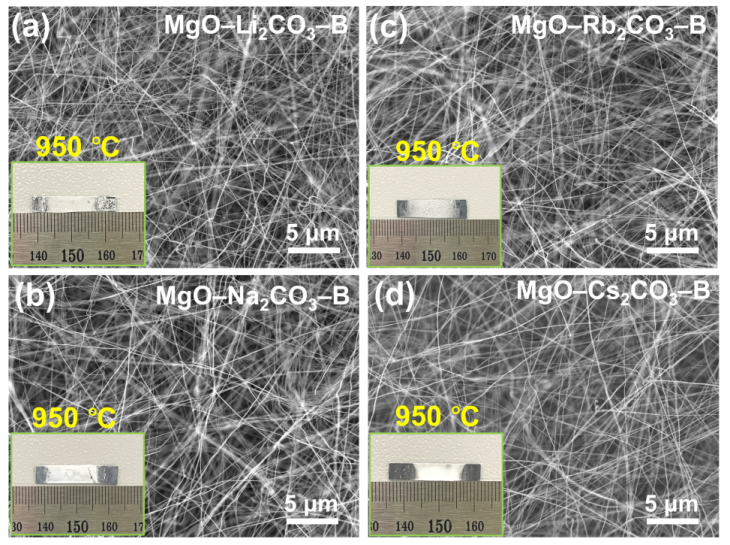


**Fig. S9** SEM images of the BNNTs grown on the SiO_2_/Si substrates from (**a**) MgO–Li_2_CO_3_–B, (**b**) MgO–Na_2_CO_3_–B, (**c**) MgO–Rb_2_CO_3_–B and (**d**) MgO–Cs_2_CO_3_–B at 950 °C


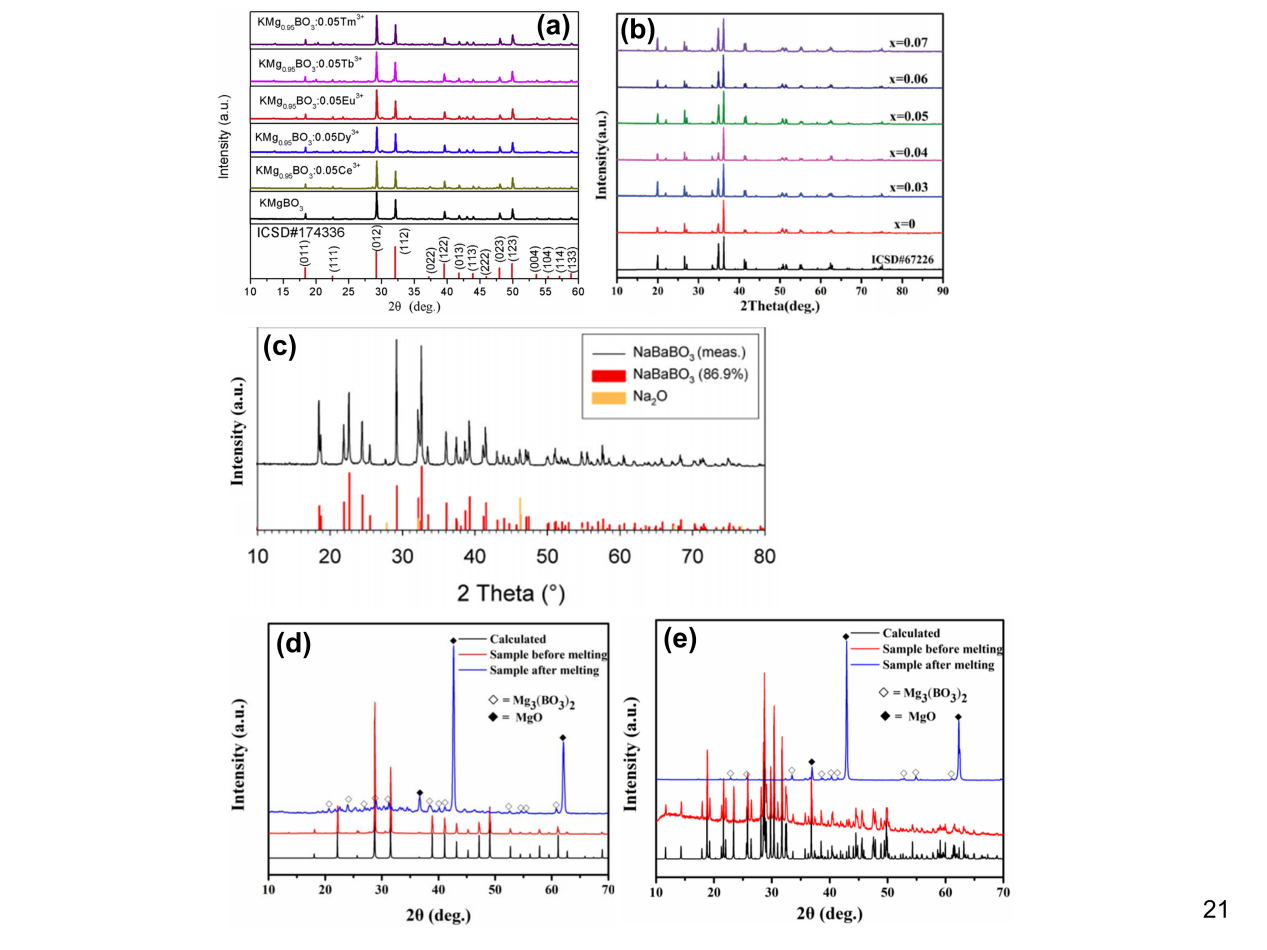


**Fig. S10** XRD patterns of (**a**) KMgBO_3_ (Reference 41 in the manuscript), (**b**) LiMgBO_3_ (Reference 53 in the manuscript), (**c**) NaMgBO_3_ (Reference 54 in the manuscript), (**d**) RbMgBO_3_ (Reference 55 in the manuscript), and (**e**) CsMgBO_3_ (Reference [55] in the manuscript). Note: All black curves in the Fig. represent standard spectra


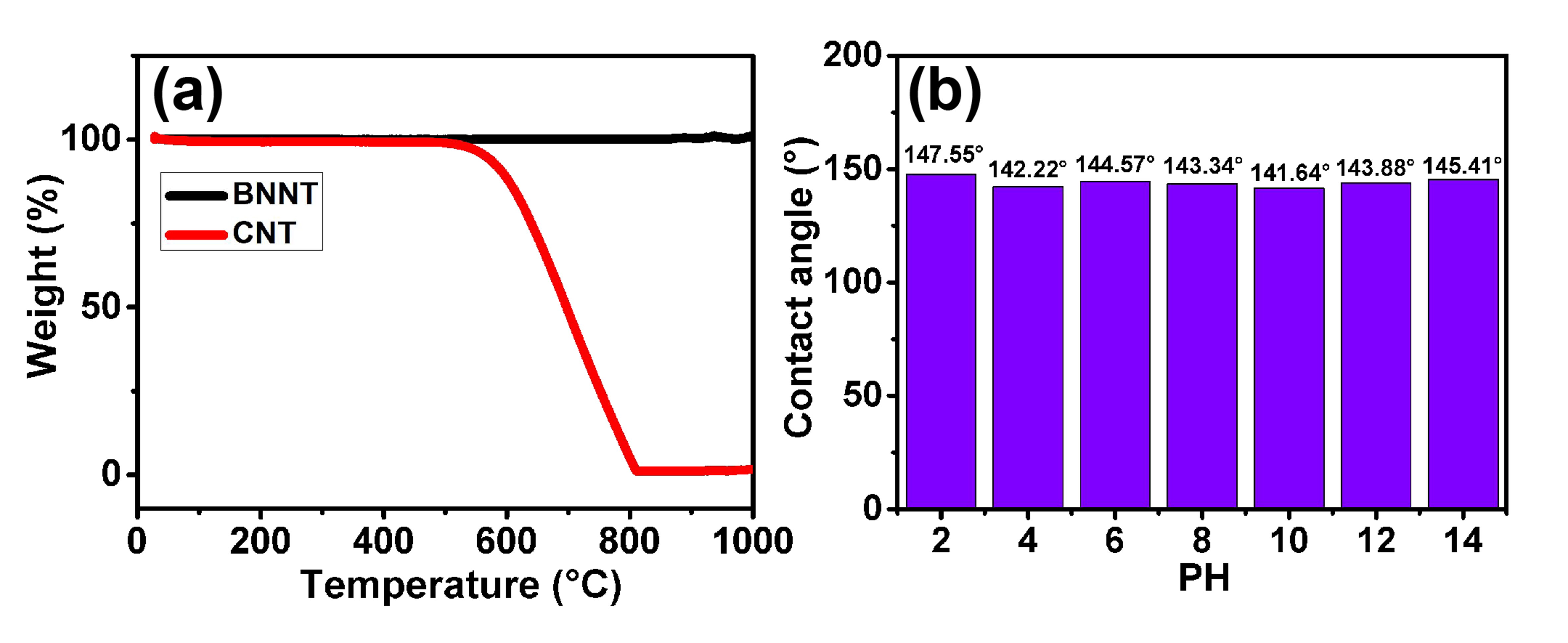


**Fig. S11** (**a**) TG graph, (**b**) Effect of variation in the pH of water droplets on the CA of water droplets on as–grown BNNT–coated SiO_2_/Si


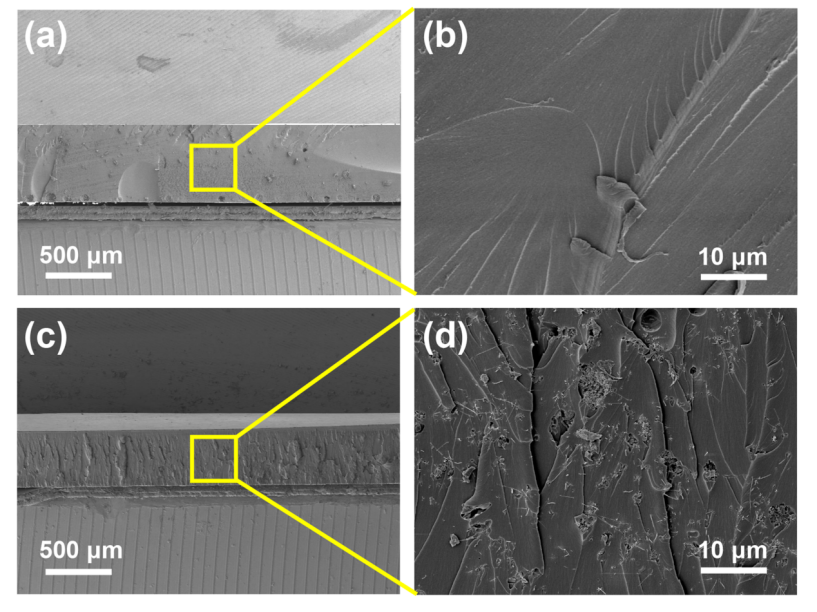


**Fig. S12** Cross–sectional SEM images of (**a, b**) a pure EP film, and (**c, d**) a BNNT/ EP composite film


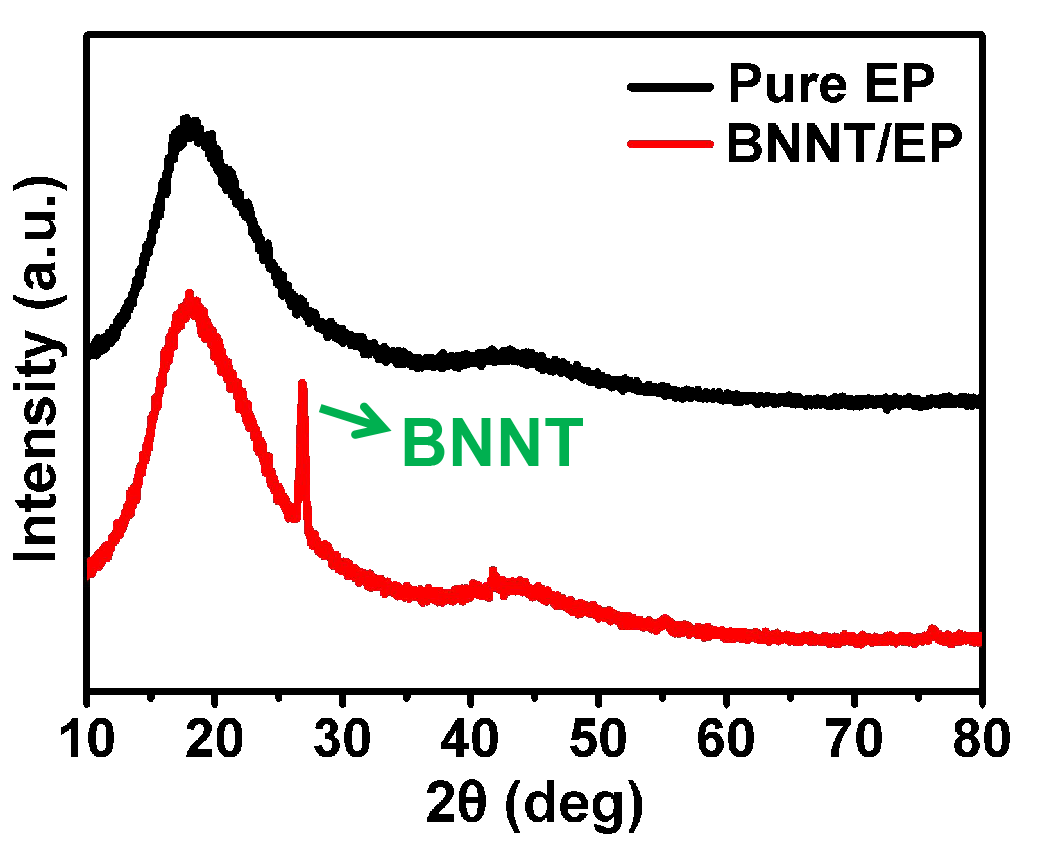


**Fig. S13** XRD patterns of pure EP film and the BNNT/EP composite film


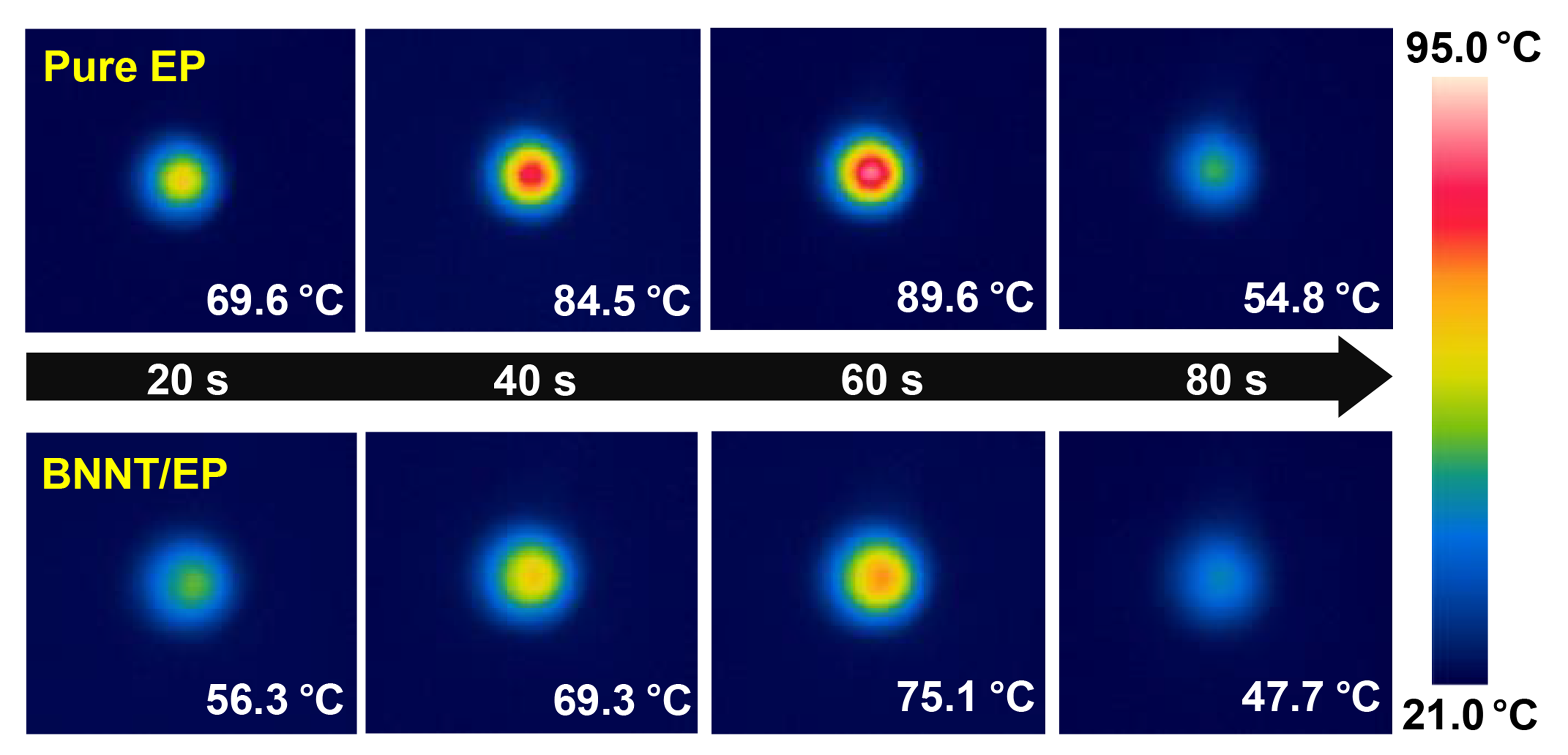


**Fig. S14** Thermal images of pure EP film and the BNNT/EP composite film

**Supplementary References**

### G. Kresse, J. Furthmu¨ller, Efficient iterative schemes for ab initio total–energy calculations using a plane–wave basis set. Phys. Rev. B. 54, 11169–11189 (1996). https://link.aps.org/doi/10.1103/PhysRevB.54.11169

# J. Perdew, K. Burke, M. Ernzerhof, Generalized Gradient Approximation Made Simple. Phys. Rev. Lett. 77, 3865–3868 (1996). https://doi.org/10.1103/PhysRevLett.77.3865

1. P. E. Blöchl, From ultrasoft pseudopotentials to the projector augmented–wave method. Projector augmented–wave method. Phys. Rev. B. **50**, 17953–17979 (1994). https://doi.org/10.1103/PhysRevB.50.17953
2. G. Kresse, D. Joubert, From ultrasoft pseudopotentials to the projector augmented–wave method. Phys. Rev. B **59**, 1758–1775 (1999). https://link.aps.org/doi/10.1103/PhysRevB.59.1758
3. A, U. Stephen, Thermochemical behavior of nepheline–forming Na–Al–Si–B–K–Li–Ca–Mg–Fe–O and hollandite–forming Ba–Cs–Ti–Cr–Al–Fe– Ga–O systems. University of South Carolina ProQuest Dissertations Publishing, 13807731 (2019). https://scholarcommons.sc.edu/etd/5153
4. A. B. Meshalkin, A. B. Kaplun, The complex investigation of the phase equilibria and melt characteristics in borate and silicate systems. J. Cryst. Growth **275**, e115–e119 (2005). https://doi.org/10.1016/j.jcrysgro.2004.10.136
